# Use of Lung Ultrasound in Reducing Radiation Exposure in Neonates with Respiratory Distress: A Quality Management Project

**DOI:** 10.3390/medicina60020308

**Published:** 2024-02-10

**Authors:** Alexandra Floriana Nemes, Adrian Ioan Toma, Vlad Dima, Sorina Crenguta Serboiu, Andreea Ioana Necula, Roxana Stoiciu, Alexandru Ioan Ulmeanu, Andreea Marinescu, Coriolan Ulmeanu

**Affiliations:** 1 Faculty of Medicine. Doctoral School, Carol Davila University of Medicine and Pharmacy, 050474 Bucharest, Romania; 2 Department of Neonatology, Life Memorial Hospital, 010719 Bucharest, Romania; 3 Faculty of Medicine, Titu Maiorescu University, 040441 Bucharest, Romania; 4 Department of Neonatology, Filantropia Hospital, 011132 Bucharest, Romania; 5 Department of Radiology, University Emergency Hospital Bucharest, 050098 Bucharest, Romania; 6 Department of Toxicology, Grigore Alexandrescu Children’s Hospital, 011743 Bucharest, Romania

**Keywords:** lung ultrasound, chest radiographs, neonatal intensive care unit

## Abstract

*Background and Objectives:* Our quality management project aims to decrease by 20% the number of neonates with respiratory distress undergoing chest radiographs as part of their diagnosis and monitoring. *Materials and Methods:* This quality management project was developed at Life Memorial Hospital, Bucharest, between 2021 and 2023. Overall, 125 patients were included in the study. The project consisted of a training phase, then an implementation phase, and the final results were measured one year after the end of the implementation phase. The imaging protocol consisted of the performance of lung ultrasounds in all the patients on CPAP (continuous positive airway pressure) or mechanical ventilation (first ultrasound at about 90 min after delivery) and the performance of chest radiographs after endotracheal intubation in any case of deterioration of the status of the patient or if such a decision was taken by the clinician. The baseline characteristics of the population were noted and compared between years 2021, 2022, and 2023. The primary outcome measures were represented by the number of X-rays performed in ventilated patients per year (including the patients on CPAP, SIMV (synchronized intermittent mandatory ventilation), IPPV (intermittent positive pressure ventilation), HFOV (high-frequency oscillatory ventilation), the number of X-rays performed per patient on CPAP/year, the number of chest X-rays performed per mechanically ventilated patient/year and the mean radiation dose/patient/year. There was no randomization of the patients for the intervention. The results were compared between the year before the project was introduced and the 2 years across which the project was implemented. *Results:* The frequency of cases in which no chest X-ray was performed was significantly higher in 2023 compared to 2022 (58.1% vs. 35.8%; *p* = 0.03) or 2021 (58.1% vs. 34.5%; *p* = 0.05) (a decrease of 22.3% in 2023 compared with 2022 and of 23.6% in 2023 compared with 2021). The frequency of cases with one chest X-ray was significantly lower in 2023 compared to 2022 (16.3% vs. 35.8%; *p* = 0.032) or 2021 (16.3% vs. 44.8%; *p* = 0.008). The mean radiation dose decreased from 5.89 Gy × cm^2^ in 2021 to 3.76 Gy × cm^2^ in 2023 (36% reduction). However, there was an increase in the number of ventilated patients with more than one X-ray (11 in 2023 versus 6 in 2021). We also noted a slight annual increase in the mean number of X-rays per patient receiving CPAP followed by mechanical ventilation (from 1.80 in 2021 to 2.33 in 2022 and then 2.50 in 2023), and there was a similar trend in the patients that received only mechanical ventilation without a statistically significant difference in these cases. *Conclusions:* The quality management project accomplished its goal by obtaining a statistically significant increase in the number of ventilated patients in which chest radiographs were not performed and also resulted in a more than 30% decrease in the radiation dose per ventilated patient. This task was accomplished mainly by increasing the number of patients on CPAP and the use only of lung ultrasound in the patients on CPAP and simple cases.

## 1. Introduction

Respiratory distress in neonates within the initial 24 h following birth is among the most prevalent neonatal conditions with the main cause of neonatal intensive care unit (NICU) admissions [1,2].

Neonatal respiratory distress (RD) arises from pulmonary immaturity and a deficit in surfactant, leading to inadequate respiratory function shortly after birth. In cases where RD worsens, the administration of surfactant is indicated based on the following criteria: (1) if FiO_2_ exceeds more than 0.3 on CPAP pressure at or above 6 cm H_2_O or (2) if lung ultrasound is suggestive of surfactant deficiency. Early surfactant administration is currently recommended, even before radiographic confirmation, based on findings from observational studies [3,4].

The diagnosis and management of neonatal lung disorders heavily rely on radiographs, and using the right radiographic approach is crucial for both patient safety and accurate diagnosis. It is important to utilize the ALARA (as low as reasonably achievable) principle when using ionizing radiation in medical imaging to minimize radiation exposure to the greatest extent possible [5,6]. In an NICU, it has been documented that estimated radiation exposure is low, ranging from 24 to 32 μGy per chest X-ray [6].

Even though a chest X-ray is usually sufficient to diagnose TTN (transient tachypnea of the newborn), RDS (respiratory distress syndrome) or congenital pneumonia, it has been shown from emerging studies that lung ultrasound has greater reliability and usefulness in various forms of respiratory distress [7,8,9].

Lung ultrasound (LU) has demonstrated its reliability in identifying infants who will need admission to the Neonatal Intensive Care Unit (NICU) due to transient neonatal tachypnea or respiratory distress syndrome as well as to predict the need for surfactant administration [10,11].

Transient Tachypnea of the Newborn (TTN) is the most common form of respiratory distress in newborns. The pathophysiological substrate is represented by the lack of absorption of fetal lung fluid, thus producing intra-alveolar exudate and interstitial edema. From an ultrasound point of view, type A lines (>3 per field) are present with the appearance of interstitial edema and in the lower lung fields a “white lung” appearance with confluent B-type lines—a pattern named a double lung point [12,13,14].

The main sonographic features of respiratory distress syndrome (RDS) involve the presence of numerous compact B-lines, leading to a “white lung” appearance, which is accompanied by a thickened and irregular pleural line. Multiple subpleural lung consolidations appear as small hypoechoic regions. It is worth noting that these patterns do not show immediate improvement even after the administration of surfactant [15,16].

Congenital pneumonia represents a frequent pathology of the newborn with a variety of sonographic findings, such as the absence of pleural line, subpleural consolidation with air bronchogram and interstitial syndrome [17].

Our quality management project has the aim to decrease by 20% the number of neonates with respiratory distress in which the chest radiograph is used in the diagnosis and monitoring of the evolution of the patient, after a period of implementation of 1 year, measured 1 year after the end of the implementation phase, compared with the year preceding the beginning of the project, by using lung ultrasound in the diagnosis and monitoring of the patients and by having clear criteria for the use of chest radiographs. By this approach, the aim is to decrease the radiation exposure of these fragile patients without affecting the diagnosis and the management of the cases.

## 2. Materials and Methods

### 2.1. Sample and Variables Analyzed

This quality management project was accomplished at Life Memorial Hospital, Bucharest. Data collection was conducted in the period between January 2021 and December 2023. A study sample of 125 patients was chosen for this research. In 2021, 29 patients were included, followed by 53 patients in 2022, and 43 patients in 2023.

The hospital’s Ethics Committee approved the quality management project before data collection.

We considered 2021 as the year of baseline (that will serve for comparison) and started to apply the method of lung ultrasound throughout 2022 and 2023 (see below for the description of the quality management program.

The inclusion criteria were admission to the Neonatal Intensive Care Unit (NICU), presence of respiratory distress, and record of treatment involving either CPAP or mechanical ventilation.

The following exclusion criteria were used: individuals with a congenital diaphragmatic hernia, necrotizing enterocolitis, or other malformations that necessitated the utilization of X-ray radiation regardless of their admission to the Neonatal Intensive Care Unit (NICU).

The clinical records of the included cases were analyzed. There were a couple of baseline variables that would be compared between the groups (years of study) to assess if the pathologies and the severity of the cases were comparable and to exclude sampling biases [18]: gestational age gender, type of delivery, treatment administered in the delivery room, type of respiratory ventilation support in the Neonatal Intensive Care Unit (NICU), pulmonary pathology, doses of administered surfactant (if any), and the presence of congenital infections

The primary outcome measures were represented by the number of ventilated patients that received X-rays each year (including the patients on CPAP, SIMV, IPPV, and HFOV), the number of X-rays performed per patient on CPAP/year, the number of chest X-rays performed per mechanically ventilated patient/year and the mean radiation dose/patient/year. The years 2021, 2022, and 2023 were compared. As the number of patients each year could not be equal, we decided to use for comparison the percent of patients receiving X-rays from the total number of patients receiving respiratory support.

As stated from the beginning, this has not been a randomized trial but rather a quality management project. We implemented a set of measures in order to decrease the number of patients. Accordingly, we compared the year 2021 when lung ultrasound was not a standard of care and 2022 and 2023 when the project has been implemented. All the ventilated (or CPAP) patients were included in the project. So, we did not have a calculated sample, but we had all the patients born during the 3-year period that needed any form of mechanical ventilation.

### 2.2. Quality Management Project Design

The resources utilized for the project were human resources and material resources.

The human resources were represented by the physicians of the neonatology unit—6 doctors—certified in neonatology; two of them were also certified in ultrasound with courses in lung ultrasound.

The neonatal unit has a functioning ultrasound machine (see below the specifications) that is available 24/7. The radiology service is also available 24/7 with qualified personnel who can perform and interpret the ultrasounds.

The first phase of the project has been a training phase with two components

- All the doctors of the units have been trained in the use and interpretation of lung ultrasound.
- A protocol for the performance of the imaging procedures has been established (Table 1)—also with training the staff for its use.

An implementation phase followed, beginning on 1 January 2022. During this phase, the patients were managed according to the new imaging protocol.

The indications for mechanical ventilation or surfactant administration were established according to the national [19] and European guidelines [4]—in case of different indications, the national guidelines have priority.

To ensure unity of practice, weekly reviews of the records of the patients with respiratory distress, lung ultrasounds and chest X-rays were performed with the staff and experts.

On 1 January 2023, we considered the transition period finished, and we followed the accomplishments of the objectives of the program.

### 2.3. Lung Ultrasound Criteria/Findings

Lung ultrasound examination was performed in all patients included in the study, using the linear probe of 8–10 MHz frequency in anterior and lateral fields, with a scanning depth of 4–5 cm adjusted to the infant’s birth weight and gestational age. The device used was a GE Vivid S60 (Manufacturer, General Electric HealthCare, Waukesha, West Milwaukee and Madison, WI, USA). To maintain the safety of the examination, the mechanical index (MI) and thermal index (TI) were maintained below 1.0.

**Figure 1:**
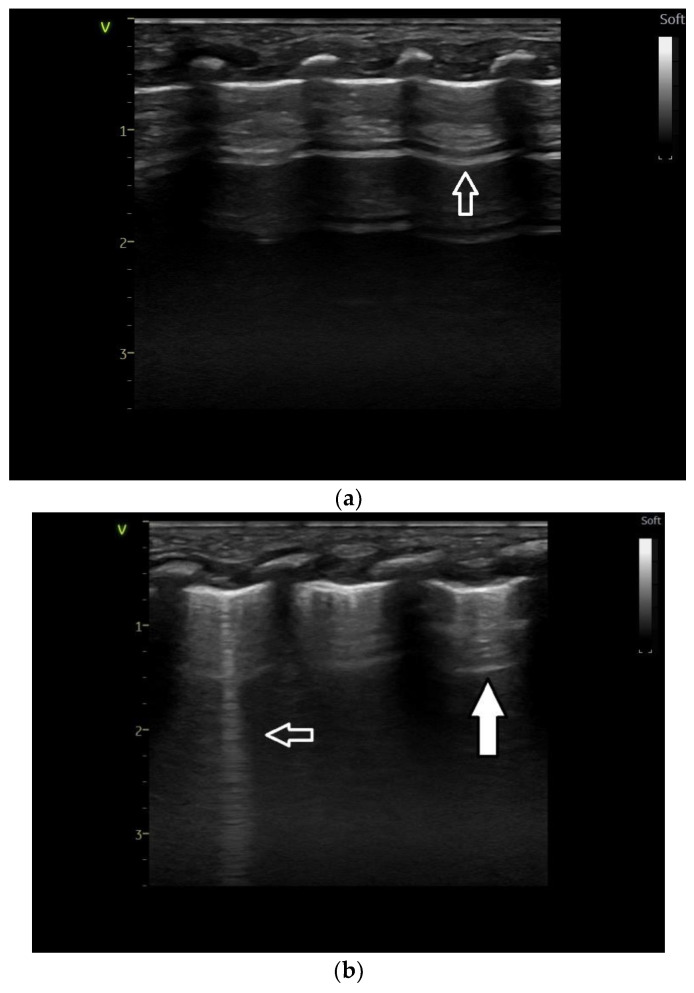
(**a**) Lung ultrasound. Normal image: A line—arrow—horizontal line parallel to the pleural line; (**b**) lung ultrasound. Normal image: A line—horizontal line parallel to the pleural line—reflection of the parietal pleura—white arrow—B line—white contour arrow—vertical line, with the origin at the visceral pleura, like a laser beam.

**Figure 2:**
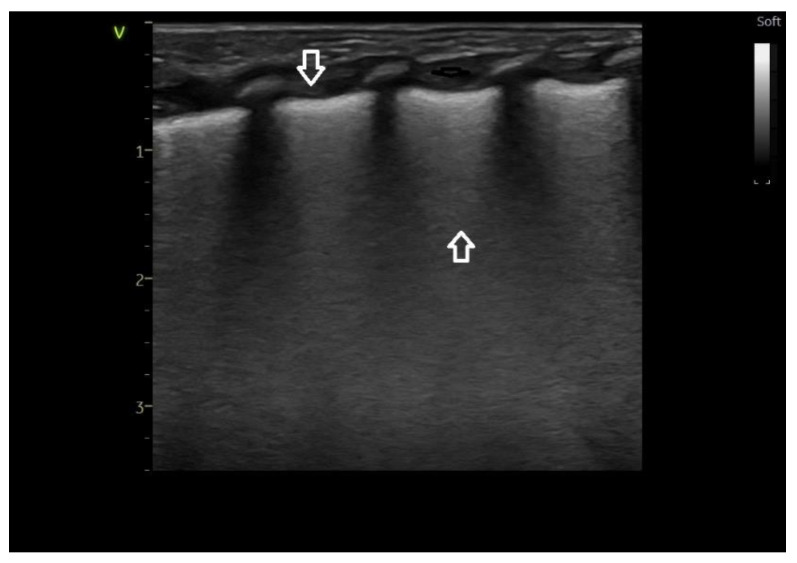
Lung ultrasound appearance of respiratory distress syndrome due to surfactant deficiency—white lung image, coalescent B-type lines occupying all lung fields—vertical arrow up; thickened pleural line–vertical downward arrow. Compare with Figure 1—normal lung.

**Figure 3:**
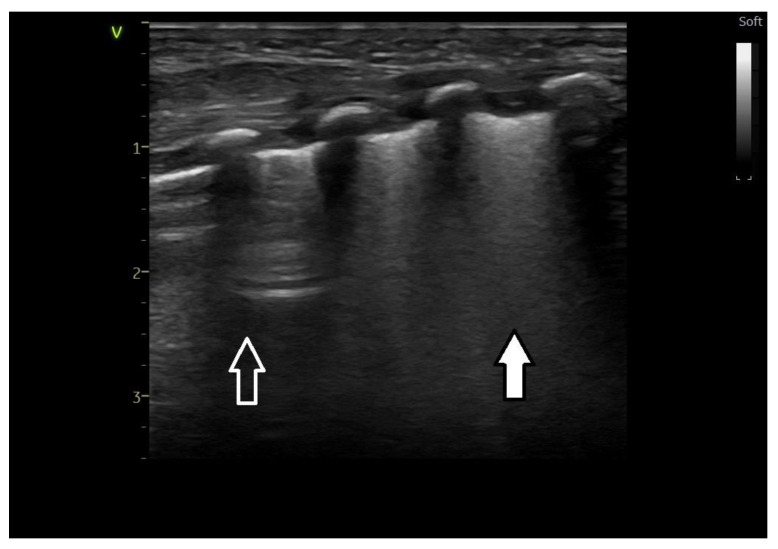
Lung ultrasound pattern in transient tachypnea of the newborn—double lung point—type A and type B lines visible in the upper lung fields—arrow with white outline; white lung appearance in the lower lung fields—white arrow. Compare with Figure 1—normal lung and Figure 2—lung ultrasound appearance in respiratory distress syndrome.

**Figure 4:**
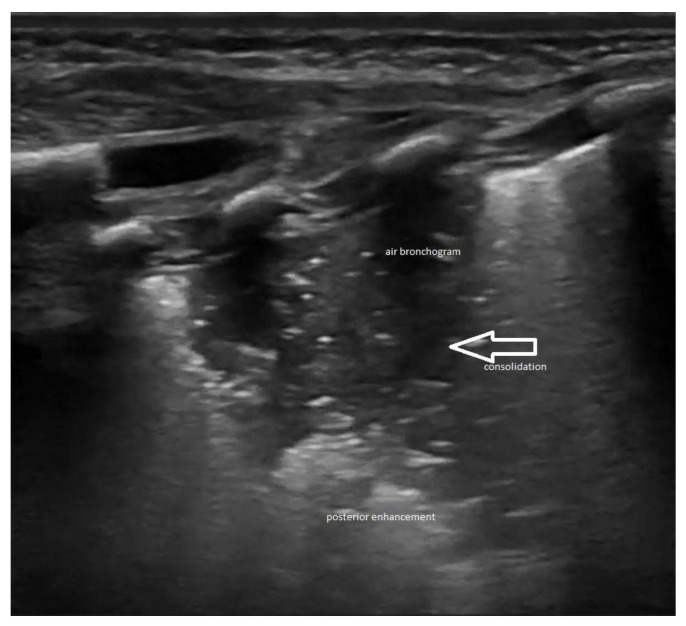
Lung ultrasound appearance in congenital pneumonia. Consolidation—arrow, air bronchogram—white spots and posterior enhancement with anfractuous appearance—shred sign.

Ultrasound images of normal lung semiology (A lines, B lines, normal lung appearance) are presented in Figure 1a,b. Typical images of ultrasound findings in lung diseases in patients in our study are shown in Figure 2, Figure 3 and Figure 4.

### 2.4. Statistics

Patient data were gathered and stored in a centralized, anonymized database. All analyses and graphical representations were performed using SPSS Statistics software (version 18.0 IBM Corporation; Armonk, NY, USA). The analysis of the correlation between the number of pulmonary X-rays and lung pathologies was performed using the one-way ANOVA test. The statistical significance for the test was chosen to be *p* < 0.05 [20].

## 3. Results

Overall, 125 patients were included in the quality management protocol from Life Memorial Hospital. The mean gestational age was 34.92 weeks (standard deviation ± 2.12) and the median was 35 weeks. In 2023, the average gestation age was significantly higher compared to that recorded in 2022 (36.14 vs. 34.92; *p* = 0.009) (Table 2).

In 2023, the average birth weight was significantly higher compared to that recorded in 2022 (2803 vs. 2504; *p* = 0.035) (Table 2).

### 3.1. Correlations by Year of Study

The characteristics of each group per year regarding the pulmonary X-ray use, applied treatment and associated pathologies can be found in Table 3.

There was a significant decrease in the proportion of males from 2021 (82.8%) to 2022 (56.6%) and 2023 (51.2%). The changes from 2021 to both 2022 and 2023 are statistically significant, indicating a notable shift in the gender distribution over the years.

There was a significant increase in the use of CPAP from 2021 (62.1%) to 2022 (83.0%). However, the slight decrease in 2023 (81.4%) was not significantly different from 2022, suggesting a stable and continuous administration of CPAP.

No statistical differences were noted in the administration of MV (mechanical ventilation) from 2021 to 2023, while in the group of patients treated with CPAP followed by MV, the percentages remained relatively stable across the three years with no significant changes. This suggests a consistent approach in using CPAP followed by ventilation mode for patients with CPAP failure over the study period.

Regarding pulmonary X-rays, the considerable decrease in the use of chest radiographs from 2021 to 2023 could be due to advancements in preventive measures, such as the implementation of lung ultrasound protocol, improvements in early treatment protocols, or changes in the prevalence of pulmonary-related conditions.

The stability in the rates of RDS and TTN suggests that there has not been a significant change in their prevalence or the effectiveness of preventive measures.

Moreover, the significant increase in 2023 of pneumothorax warrants attention. It could suggest changes in clinical practices, an increase in predisposing factors, or variations in reporting or diagnostic criteria. The frequency of cases in which no pulmonary X-rays were performed was significantly higher in 2023 compared to 2022 (58.1% vs. 35.8%; *p* = 0.03) or 2021 (58.1% vs. 34.5%; *p* = 0.05). The frequency of cases with pulmonary X-rays was significantly lower in 2023 compared to 2022 (16.3% vs. 35.8%; *p* = 0.032) or 2021 (16.3% vs. 44.8%; *p* = 0.008) (Table 4).

### 3.2. Correlations between X-rays and Categories of Treatment Applied to Patients

The basic characteristics and the pathology encountered were analyzed separately for the subgroups of patients treated with CPAP (Table 5), CPAP followed by MV (Table 6) and MV (Table 7).

The mean number of X-rays per CPAP patient was lowest in 2023 (0.57) and highest in 2022 (1.02). However, the overall *p*-value of 0.208 suggests that these differences across the years are not statistically significant.

The wide range of standard deviations each year indicates variability in the number of X-rays per patient.

The total analysis over the three years shows a mean of 0.79 X-rays per patient, with a 95% confidence interval ranging from 0.56 to 1.03, reflecting the average tendency across the study period.

The absence of statistically significant differences year over year suggests that the variation in the number of X-rays per CPAP patient may be attributed to the learning curve of decreasing the use of chest X-rays. These results can be useful for understanding the typical radiographic imaging needs of CPAP patients and for assessing the consistency of X-ray usage in CPAP treatment over the specified period.

The slight annual increase in the mean number of X-rays (from 1.80 in 2021 to 2.33 in 2022, and then 2.50 in 2023) could indicate a trend toward more frequent use of X-rays in managing patients who require both CPAP and VM.

The high standard deviations, especially in 2022 and 2023, point to significant variability in the number of X-rays per patient. This variability might reflect differences in individual patient conditions, the complexity of cases, or varied clinical judgments about the necessity for X-ray imaging.

The total analysis over the three years shows a mean of 2.25 X-rays per patient, with a 95% confidence interval ranging from 1.63 to 2.87, indicating a general trend toward a higher number of X-rays for patients undergoing CPAP followed by VM compared to CPAP alone.

There is a noticeable increase in the mean number of X-rays per ventilated patient from 2021 (1.50) to 2022 (2.11) and 2023 (2.21). This suggests a potential upward trend in the use of X-rays in ventilated patients over these years.

The wide range of standard deviations indicates high variability in the number of X-rays per patient each year, suggesting individualized care and differences in patient conditions or treatment protocols.

The analysis over the three years shows a mean of 1.94 X-rays per patient, indicating an overall tendency toward nearly two X-rays per ventilated patient on average.

Regarding the radiation dose received per year, we used a mean dose of 9 Gy × cm^2^ and analyzed the following data presented in Table 8.

Considering 2022 as the comparison year, 2021 had a 57.57% rate of radiation dose/patient, while 2023 had a reduction of 54.54% after using lung ultrasound concomitantly with pulmonary X-rays. There has been a reduction in the mean radiation dose per patient from 5.89 Gy × cm^2^ in 2021 to 3.76 Gy × cm^2^ in 2023 (a 36% reduction).

The number of cases increased significantly from 2021 to 2022, and then decreased in 2023, but it remained higher than in 2021. This might reflect changes in the number of patients undergoing pulmonary X-rays or changes in lung ultrasound practices.

## 4. Discussion

Our quality management project accomplished its goal, i.e., to decrease the number of neonates that received respiratory support (mechanical ventilation or CPAP) in which chest X-rays were performed by more than 20% compared with the year before the implementation of the project.

As such a project, randomization and isolation of the intervention in order to assess its efficacy were not entirely possible. As a consequence, we cannot state certainly that this reduction has been related solely to the use of ultrasound. Instead, we noticed an increase in the use of CPAP in the patients in the need for respiratory support between 2021 and 2022 and, since we performed less chest X-rays in the patients on CPAP, this could have been a contributing factor. Another confounding factor could have been the difference in gestational age between 2022 and 2023 (but not between 2021 and 2023), but the main difference in the percent of patients receiving chest X-rays has been between 2021 and 2023 (in which case, no significant difference in the gestational ages was noticed). Another factor could have been the incidence of the different respiratory conditions (pneumonia, transient tachypnea of the newborn, respiratory distress syndrome), but in these cases there was no noted difference between the years. In conclusion, we consider that the main factor that could be related to a reduction in the percent of X-rays performed, together with the more widespread use of lung ultrasound, could be the more frequent use of CPAP as the only respiratory support technique.

We consider the strong points of the study to be the implementation of a lung ultrasound teaching program for all the doctors in the NICU and the study design as a quality management protocol. During the first year (2022), there was a learning curve in the use of both LU and chest X-rays with a decrease in pulmonary radiographs in 2022 and 2023. We showed that it is possible, through teaching and the judicious use of lung ultrasound, to decrease the number of patients that were exposed to X-rays, especially in the case of patients on CPAP.

Our choice for lung ultrasound has been supported by the ease of using the method and a good diagnostic value for the neonatal lung pathology [21,22]. Lung ultrasound (LUS) presents distinct signs linked to respiratory distress, which can be better identified by observing the lung tissue in real time and conducting multiple ultrasounds across the lung fields in various planes [21]. It is important to note that ultrasound is known for being influenced by the operator’s skill, which can introduce potential errors. However, adhering to a standardized approach can reduce operator variability and enhance diagnostic precision [22].

While there have been concerns about excessive radiation exposure in NICU radiographic examinations, the research reviewed consistently found that the radiation levels in most individual radiographic procedures are minimal in modern NICUs [23,24]. Nonetheless, it remains crucial to adhere to the ALARA (“as low as reasonably achievable”) principle, because NICU patients are exceptionally susceptible to the long-term cumulative effects of radiation exposure throughout their lives [25,26,27]. A study from Gao that analyzed 1381 patients admitted to the NICU over three years showed that lung ultrasound is completely reliable for diagnosis and lung disease differential diagnosis and could potentially replace pulmonary X-ray use [9].

Previously published papers have shown that neonatologists can attain a high level of proficiency in lung ultrasound following training programs of 2 days or more (either on site or combined with e-learning) [28]. As a result, the use of pulmonary X-rays has decreased, and more focus has been put on preventive management therapies as well as ultrasound-guided surfactant therapy [28,29].

Considering the potential risks associated with ultrasound examinations, it is important to recognize that while ultrasound is generally considered less harmful than other imaging techniques like radiology or CT scans, it still carries real biological risks that should be considered [12,15,30]. Ultrasound examinations can induce thermal effects, such as overheating and mechanical effects, particularly cavitation, on living tissues. If these effects surpass specific thresholds, they have the potential to harm living tissues [12,25,30].

We are aware also of certain weak points and limitations of the project. Two of them are represented by the monocentric design of the study and the fact that there has not been a uniform approach to the indications for X-rays, allowing freedom for the clinician in the decision to perform this procedure. This approach could also be considered a strong point, because we left the clinicians’ choice in order to establish the diagnosis modality for the patient, trying to decrease the risk of under-diagnosing certain pathologies and harming the patient by not performing an X-ray when needed. It is our belief, though, that positive results (decreasing the use of X-rays) could be obtained better by education than by constraint and regulations and that results obtained this way will be more durable and safer for the patients. The monocentric character of the project lead to a smaller number of patients included. We consider though that the lessons learned in this project (see below) could provide us with more experience for a multicentric approach with the same goal.

Another limitation could be considered the fact that the patients included were not of small gestational ages (not less than 30 weeks GA). This could be also an issue for a future research or quality management project.

A notable issue in our quality management project surfaced when, despite a reduction in the overall number of X-rays, there was a rising trend in the number of X-rays administered per mechanically ventilated patient. The fact that we did not obtain the expected results in the case of the mechanically ventilated patients suggested the topic of a future direction of quality management research project, in which, by education and support for the staff, we will aim for a decrease in the use of chest radiographs in this category of patients. Looking ahead, our objective is to explore in greater detail the factors underlying the increased frequency of X-rays for individual patients, which is likely linked to inherent variations. We aim to deepen our understanding and refine the number of X-rays conducted. Our future strategy involves standardizing the utilization of X-rays and implementing the pulmonary X-ray protocol. This shift ensures that decisions regarding X-ray procedures are no longer solely reliant on physician opinions.

An Italian panel from 10 centers has shown that their guidelines outline specific criteria aimed at maintaining a superior level of neonatal care within NICUs. These criteria encompass various aspects such as protocols for procedures, facility requirements, recommended equipment, quality control measures, radiation protection protocols for both infants and staff and communication regarding radiation-related risks. This approach aims to establish a standardized framework for managing exposures in NICUs while still allowing for adaptability and flexibility as needed [31].

Another limitation could have been that we did not use other imaging modalities to compare the results with LU. Other imaging modalities are used more and more in the diagnosis and appreciation of prognosis of lung diseases in the neonate [32,33,34,35]. Of these, lung MRI, both fetal and neonatal, is worth mentioning.

Regarding fetal MRI, it is tempting to try to use this for establishing the prognosis in the lung conditions in the newborn. However, in a review published this year, the studies using fetal MRI for prognosis were used in most of the cases in patients with congenital diaphragmatic hernia and in a small number of patients with congenital lung malformations [30]. The indication for fetal MRI, as also mentioned in this paper, is pulmonary hypoplasia [30], so this is not the case in the premature infants with respiratory distress that do not have hypoplastic lungs (like the patients with diaphragmatic hernia) but rather lungs deficient in the production of surfactant (RDS) or with delayed absorption of lung fluid (TTN) or meconium aspiration or pneumonia. The same review concludes that for the moment, the prognostic value depends on the volumetric assessment and that the prediction of postnatal respiratory outcome could be feasible if emerging techniques could be employed and validated in pathological lungs [32]. This could be, though, a future direction of study.

Also, a future direction of study could be the use of neonatal lung MRI, now a very promising imaging technique, especially with ultrafast sequences and short exposure times and the availability of newer portable techniques [33,34,35]. Feasibility studies have shown the possibility of using this method in the NICU [33].

There are, although, several points to be discussed regarding neonatal lung MRI:

- The studies show good correlations between MRI patterns and chronic conditions like bronchopulmonary dysplasia [33,34,35] but do not mention the use of the method in the acute setting like respiratory distress syndrome and transient tachypnea of the newborn.
- The cost of an MRI in the NICU is far greater than the cost of a MRI, and this is not a technique to be used yet in all the neonates.
- The aim of our project has been to decrease the use of the X-rays by using simple diagnostic and assessment methods, like clinical examination, history, blood gases and ultrasound, and MRI is obviously not a simple technique to be used in all the settings.
- Although MRI is a promising and very accurate technique for the assessment of neonatal lungs, it is not yet passed into usual clinical practice, and it is not mentioned in the RDS guidelines like X-rays and ultrasound. Accordingly, since our goal has been to provide a model to be simple and replicated in all the units, we did not include lung MRI in our assessment protocol.

We would though consider in the future the use of MRI in a smaller group of patients, as the other studies did, in order to demonstrate the value of this technique.

## 5. Conclusions

The quality management project accomplished its goal by resulting in a statistically significant increase in the number of ventilated patients in which chest radiographs were not performed and also resulted in a more than 30% decrease in the radiation dose per ventilated patient. This task was accomplished mainly by increasing the number of patients on CPAP and the use only of lung ultrasound in the patients on CPAP and in simple cases.

As we did not perform a study with a control group, we cannot say for certain that this reduction was the result of only the use of lung ultrasound. Instead, we could consider that a bundle of care has been applied, consisting of the increased use of CPAP, increased use of lung ultrasound and more awareness to the indications of the X-rays.

The detection of more frequent use of chest radiographs in the case of more severe cases suggested to our group that the next quality management goal in the appropriate use of chest radiographs will be the introduction of more restrictive criteria and the improvement of the skills of the staff in using the lung ultrasound not only in the monitoring of the premature infants with respiratory distress syndrome or transient tachypnea of the newborn but also in the monitoring of all the categories of ventilated neonates.

## Figures and Tables

**Table 1:** Imaging procedures protocol in patients with respiratory distress.

Lung ultrasound - Performed in all the patients 60–90 min after delivery - Performed as needed in the case of ventilated patients or patients on CPAP * Indications for chest radiographs (X-rays) - After intubation, check the status of the patient and the position of the ET tube - At any deterioration of the clinical status of a ventilated patient - For confirming the diagnosis of a pneumothorax - As decided by the clinicians’ clinical judgement **

* Lung ultrasound performed by the bedside clinician. In case of doubt, the images were sent electronically to the expert for a second opinion. ** The attending physician had the freedom to decide to perform a chest X-ray in any case of a patient with respiratory distress not mentioned above, based on their clinical judgement, but had to state the reason in the patient’s record.

**Table 2:** Descriptive data of gestational age and birth weight (g) compared by study years.

Parameters	Year of Study			One-Way ANOVA Test
	2021	2022 *	2023 **	
Gestational age				
mean ± SD	35.55 ± 1.80	34.92 ± 2.12 ^d^	36.14 ± 11.87 ^d,b^	**0.012**
median	35	35	36	
limits	32–39	30–39	30–39	
Birth weight				
mean ± SD	2680 ± 562.56	2504 ± 621.79 ^d^	2803 ± 535.10 ^d,c^	**0.043**
median	2670	2420	2890	
limits	1230–3500	1410–7592	1500–3670	

One-way ANOVA test post hoc Tukey HSD * 2022 vs. 2021; ** 2023 vs. 2021 and 2023 vs. 2022. ^b^ *p* < 0.01; ^c^ *p* < 0.05; ^d^ *p* > 0.05.

**Table 3:** Comparative clinical data by year of study.

Parameters	Year of Study						Chi-Square Test Likelihood Ratio
	2021 (*n* = 29)	2022 (*n* = 53)	*p* Value for Chi2 Test 2022 vs. *2021*	2023 (*n* = 43)	*p* Value for Chi2 Test 2023 vs. *2021*	*p* Value for Chi2 Test 2023 vs. *2022*	
Male	24 (82.8%)	30 (56.6%)	**0.018**	22 (51.2%)	**0.007**	0.597	**0.013**
CPAP	18 (62.1%)	44 (83.0%)	**0.036**	35 (81.4%)	0.070	0.837	0.087
VM	16 (55.2%)	18 (34.0%)	0.064	14 (32.6%)	0.058	0.885	0.110
CPAP followed by VM	5 (17.2%)	9 (17.0%)	0.976	6 (14.0%)	0.706	0.686	0.901
Pulmonary Rx	19 (65.5%)	33 (62.3%)	0.771	18 (41.9%)	**0.050**	**0.048**	0.067
**Associated pathologies**							
SDR	21 (72.4%)	44 (83.0%)	0.260	31 (72.1%)	0.976	0.200	0.359
TTN	5 (17.2%)	8 (15.1%)	0.800	7 (16.3%)	0.915	0.874	0.967
Congenital pneumonia	3 (10.3%)	1 (1.9%)	0.091	0 (0%)	**0.032**	0.368	**0.045**
Meconium aspiration	0 (0%)	1 (1.9%)	0.459	1 (2.3%)	0.412	0.882	0.580
Pneumothorax	1 (3.4%)	0 (0%)	0.176	5 (11.6%)	0.221	**0.011**	**0.014**

**Table 4:** Descriptive data of comparative pulmonary Rx number by study years.

Lung Rx Number	2021 (*n* = 29)	2022 * (*n* = 53)	2023 ** (*n* = 43)	Chi-Square Test Likelihood Ratio
0	10 (34.5%)	19 (35.8%) ^d^	25 (58.1%) ^c,c^	0.146
1	13 (44.8%)	19 (35.8%) ^d^	7 (16.3%) ^b,c^	
2	4 (13.8%)	8 (15.1%) ^d^	5 (11.6%) ^d,d^	
3	2 (6.9%)	3 (5.7%) ^d^	5 (11.6%) ^d,d^	
≥4	0 (0.0%)	4 (7.6%) ^d^	1 (2.3%) ^d,d^	

Chi2 test * 2022 vs. 2021; ** 2023 vs. 2021 and 2023 vs. 2022. ^b^ *p* < 0.01; ^c^ *p* < 0.05; ^d^ *p* > 0.05.

**Table 5:** Number of X-rays/patient CPAP. *p* = 0.208.

	*N*	Mean	Std. Deviation	Std. Error	95% Confidence Interval for Mean		Minimum	Maximum
					Lower Bound	Upper Bound		
2021	18	0.67	0.907	0.214	0.22	1.12	0	3
2022	44	1.02	1.320	0.199	0.62	1.42	0	6
2023	35	0.57	1.065	0.180	0.21	0.94	0	4
Total	97	0.79	1.172	0.119	0.56	1.03	0	6

**Table 6:** Number of X-rays/patient CPAP followed by MV. *p* = 0.688.

	*N*	Mean	Std. Deviation	Std. Error	95% Confidence Interval for Mean		Minimum	Maximum
					Lower Bound	Upper Bound		
2021	5	1.80	0.837	0.374	0.76	2.84	1	3
2022	9	2.33	1.732	0.577	1.00	3.66	1	6
2023	6	2.50	1.049	0.428	1.40	3.60	1	4
Total	20	2.25	1.333	0.298	1.63	2.87	1	6

**Table 7:** Number of X-rays/ventilated patient. *p* = 0.155.

	*N*	Mean	Std. Deviation	Std. Error	95% Confidence Interval for Mean		Minimum	Maximum
					Lower Bound	Upper Bound		
2021	16	1.50	0.730	0.183	1.11	1.89	1	3
2022	18	2.11	1.410	0.332	1.41	2.81	1	6
2023	14	2.21	0.975	0.261	1.65	2.78	1	4
Total	48	1.94	1.119	0.161	1.61	2.26	1	6

**Table 8:** Radiation dose per year.

Parameter	2021	2022	2023
*N*	29	53	43
Pulmonary X-ray	19	33	18
Radiation dose (mean = 9 Gy × cm^2^)	171	297	162
Radiation dose/ventilated patient (including CPAP)	5.89	5.6	3.76

## Data Availability

The database of the study can be accessed upon request at the address adrian.toma@prof.utm.ro.

## Abbreviations

ANOVA	analysis of variance
CPAP	continuous positive airway pressure
ET tube	endotracheal tube
Gy	Gray
HFOV	High-frequency oscillatory ventilation
H_2_O	water
IPPV	intermittent positive pressure ventilation
LU	lung ultrasound
MHz	megahertz
N	number
NICU	neonatal intensive care unit
NY	New York
RD	respiratory distress
RDS	respiratory distress syndrome
SIMV	synchronized intermittent mandatory ventilation
TTN	transient tachypnea of the newborn
WI	Wisconsin
Vs	versus
X-rays	radiograph
